# NUTRALYS^®^ pea protein: characterization of *in vitro* gastric digestion and *in vivo* gastrointestinal peptide responses relevant to satiety

**DOI:** 10.3402/fnr.v59.25622

**Published:** 2015-04-13

**Authors:** Joost Overduin, Laetitia Guérin-Deremaux, Daniel Wils, Tim T. Lambers

**Affiliations:** 1 Department of Health, NIZO Food Research, Ede, The Netherlands; 2 Department of Biology and Nutrition, Roquette Frères, Lestrem, France; 3 Nutrition Management, Roquette Frères, Lestrem, France

**Keywords:** pea protein, dairy proteins, satiety, gastrointestinal peptides, *in vitro* gastric digestion

## Abstract

**Background:**

Pea protein (from *Pisum sativum*) is under consideration as a sustainable, satiety-inducing food ingredient.

**Objective:**

In the current study, pea-protein-induced physiological signals relevant to satiety were characterized *in vitro* via gastric digestion kinetics and *in vivo* by monitoring post-meal gastrointestinal hormonal responses in rats.

**Design:**

Under *in vitro* simulated gastric conditions, the digestion of NUTRALYS^®^ pea protein was compared to that of two dairy proteins, slow-digestible casein and fast-digestible whey. *In vivo*, blood glucose and gastrointestinal hormonal (insulin, ghrelin, cholecystokinin [CCK], glucagon-like peptide 1 [GLP-1], and peptide YY [PYY]) responses were monitored in nine male Wistar rats following isocaloric (11 kcal) meals containing 35 energy% of either NUTRALYS^®^ pea protein, whey protein, or carbohydrate (non-protein).

**Results:**

*In vitro*, pea protein transiently aggregated into particles, whereas casein formed a more enduring protein network and whey protein remained dissolved. Pea-protein particle size ranged from 50 to 500 µm, well below the 2 mm threshold for gastric retention in humans. *In vivo*, pea-protein and whey-protein meals induced comparable responses for CCK, GLP-1, and PYY, that is, the anorexigenic hormones. Pea protein induced weaker initial, but equal 3-h integrated ghrelin and insulin responses than whey protein, possibly due to the slower gastric breakdown of pea protein observed *in vitro*. Two hours after meals, CCK levels were more elevated in the case of protein meals compared to that of non-protein meals.

**Conclusions:**

These results indicate that 1) pea protein transiently aggregates in the stomach and has an intermediately fast intestinal bioavailability in between that of whey and casein; 2) pea-protein- and dairy-protein-containing meals were comparably efficacious in triggering gastrointestinal satiety signals.

Adjustment of dietary composition is a sensible approach to control energy balance and address the global problem of overweight and associated health problems (1). Laboratory and intervention studies indicate that increasing dietary protein levels may aid weight control via several mechanisms, such as enhanced within-meal satiation and between-meal satiety, stimulation of post-meal energy expenditure and conservation of metabolically active lean body mass during reduced-calorie diets (2, 3). Moreover, post-ingestive physiological signals and effects on body weight vary among different types of dietary protein (4–7) . Casein and whey, the two major fractions of bovine milk protein illustrate this point. Casein has been dubbed a ‘slow’ protein by Boirie et al. (8) because its molecules aggregate to form a larger protein network within the acidic stomach *milieu*, in a process that delays nutrient entry to the small intestine, resulting in the small-intestinal absorption of casein's amino acids and their appearance in the circulation (5). In contrast, the faster-digestible whey protein does not aggregate within the stomach, enter the small intestine more rapidly after ingestion, and induce faster and briefer effects on protein metabolism (8, 9). Corresponding to the different gastric processing of casein and whey, different time courses of intestinal satiety signals, reported satiety, and food intake have been reported for these two proteins (10), with results varying according to the exact study design (11, 12).

Assessment of digestibility and physiological effects of proteins is becoming increasingly relevant as novel dietary protein sources are being explored, driven by food technological advances, changing consumer awareness, and factors related to cost and sustainability (13). While the amino-acid composition of a protein defines its nutritional value (14, 15), it does not fully predict the intensity of the post-ingestive physiological signals relevant to satiety. This underscores the importance of measuring the kinetics of gastrointestinal digestion of proteins and gastrointestinal hormonal responses to ingested proteins. Specifically, protein ingestion induces a plasma rise of anorexigenic gastrointestinal hormones cholecystokinin (CCK), glucagon-like peptide 1 (GLP-1), and peptide YY (PYY), and a suppression of the orexigenic stomach hormone, ghrelin. These effects are considered to be physiological markers of within-meal satiation and post-meal satiety. Proteins also stimulate the release of insulin and leptin, both of which may affect long-term energy balance via pathways in the central nervous system. The present studies were undertaken to elucidate gastrointestinal digestion and hormonal effects of pea protein. Pea protein is currently being developed by the food industry for meat, dairy, and vegetarian products, for high-protein foods such as in sports nutrition. Several studies have demonstrated that pea protein may stimulate satiety-related signaling and behavior in rodents and humans. Significant peptide release has been observed in cultured enteroendocrine cells or murine intestinal tissues exposed to whole pea protein and pea protein hydrolysate (16). Pre-meal gastric infusions of pea protein in rats reduce meal size (17). In humans, intestinal infusion and oral ingestion of pea protein induces satiety in normal-weight and obese subjects (18, 19).

The specific aim of the current *in vitro* and *in vivo* studies was to characterize satiety-related, digestion and hormonal effects of purified, consumer-grade pea protein (NUTRALYS^®^, Roquette Frères, Lestrem, France). Using an *in vitro* model of stomach digestion, equipped with in-line rheological measurements, effects of pea protein were compared with the fast-digestible protein whey and slowly digestible casein as reference proteins. The *in vivo* hormonal test compared effects of isocaloric, mixed-macronutrient meals enriched in pea protein, whey protein, or carbohydrate (no protein). Whey protein was included as a positive control because of its well-documented modulation of satiation and energy intake in rats and humans (19).

## Materials and methods

### Protein ingredients used in the *in vitro* and *in vivo* studies

NUTRALYS^®^ pea protein is extracted from yellow peas (*Pisum sativum*) as follows. First, peas are cleaned and ground to a dry flour. The flour is then hydrated and the pea starch and internal fiber are extracted separately. The protein fraction is coagulated for further purification and carefully dried in a multi-stage spray dryer. The resulting purified pea-protein isolate contains 85% protein, 7% fat, 3% carbohydrate, and 5% ash on a dry matter basis. Whey Protein (BiPRO WPI, Davisco Foods International, Le Sueur, MN, USA) is isolated from bovine milk as the fraction of proteins that remain soluble at acidic conditions (pH 4.6). Further isolation and concentration of proteins is achieved via ion-exchange chromatography and microfiltration, yielding 97–98% undenatured protein on a dry-matter basis. The amino-acid profiles of pea and whey protein are depicted in Table 1. Casein (sodium caseinate, Caldic Ingredients B.V., Oudewater, the Netherlands) is produced from skimmed milk by letting acid (pH=4.6)-precipitated casein, 80% of the protein source in milk, react with sodium hydroxide. The product is then drum- or spray-dried, yielding a powder with a 95% protein content on a dry-matter basis.

**Table 1:** Amino acid profiles (w/w% of total protein) of NUTRALYS^®^ pea protein and BiPRO^®^ whey protein

	NUTRALYS^®^ pea protein^a^	BiPRO^®^ whey protein^b^
Alanine	4.3	4.5
Arginine	8.7	2.5
Aspartic acid	11.5	11.1
Glutamic acid	16.7	16.6
Histidine	2.5	2.0
Isoleucine	4.7	5.5
Leucine	8.2	12.1
Lysine	7.1	11.1
Methionine	1.1	2.5
Phenylalanine	5.5	3.5
Proline	4.3	4.5
Serine	5.1	3.0
Threonine	3.8	4.5
Tryptophan	1.0	3.0
Valine	5.0	5.5

a Roquette, Lestrem, France.

b Davisco Foods, Le Sueur, MN, USA.

### *In vitro* simulation of gastric digestion of proteins

*In vitro* models of gastrointestinal digestion provide a controlled approach to compare foods in terms of their physical and chemical behaviors in stomach and intestines (20). Three-percent protein solutions of NUTRALYS^®^ pea protein, whey protein, or casein were submitted to NIZO SIMPHYD, an *in vitro* model of gastric digestive conditions, combined with in-line viscosity measurements. In short, viscosity measurements were performed using a controlled stress rheometer (AR-2000; TA Instruments, New Castle, DE, USA) equipped with a stainless-steel vane geometry (stator inner radius 26.85 mm; rotor outer diameter 24.5 mm, height, 73 mm). Protein samples were tested under steady shear conditions (continuous ramp, 37°C, 3 h at 75 s^−1^). After an initial 5 min baseline exposure and measurements, simulated gastric acidification from initial pH levels (6.5–7.0) toward pH 1.5–2 was started while viscosity was monitored continuously. Upon reaching a pH of between 1.5 and 2, which typically occurred within 15 min, gastric enzymes pepsin (Sigma-Aldrich, St. Louis, MO, USA) and lipase (Amano Enzyme Europe Ltd, Chipping Norton, UK), were added to the medium and viscosity was monitored until 2 h after the onset of the procedure. Particle size distribution was measured off-line using a Malvern Mastersizer 2000 (Malvern Instruments Ltd., Malvern, UK).

### *In vivo* gastrointestinal hormone responses to protein meals

#### Animals and diets

Twelve male SPF Wistar rats (aged 14–16 weeks; weight 290–360 g) were housed individually at constant room temperature (20–22°C) and relative humidity (50–60%), and exposed to a 12 h/12 h light/dark cycle. All animal procedures were submitted to and approved by the institutional animal use and care committee of Wageningen University (WUR-DEC protocol #2009006.c). The animals had free access to water and regular rodent chow following the recommendations of the American Institute of Nutrition (21). The three test foods were presented as liquid emulsions with an equal energy density (1.1 kcal/g). To ensure detection of differential effects of pea and whey proteins, meals were designed to have a high protein (35 kcal%) content. In the non-protein test food, protein was replaced by an isocaloric quantity of sucrose (BDH, VWR, Amsterdam, the Netherlands). The energy composition of the foods (in kcal%) was as follows: pea-protein food (pea protein [NUTRALYS^®^ pea protein, Roquette] 35%, sucrose 35%, corn oil 30%); whey-protein food (whey protein [BiPRO WPI, Davisco Foods International, MN, USA] 35%, sucrose 35%, corn oil 30%) and non-protein control food (sucrose 70%, corn oil 30%). Gum arabic (3.7 w/w%) was added as an emulsifier. The viscosities of the test foods were equalized by addition of 0.2 or 0.4 w/w% of xanthan gum (Sigma-Aldrich), a non-caloric thickening agent, which resists gastrointestinal digestion, does not aggregate or otherwise solidify during digestion and has a negligible effect on blood glucose or insulin (22).

#### Procedure

**Fig. 1:**
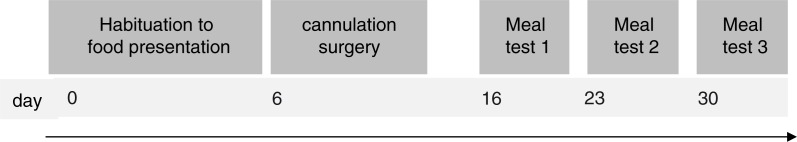
Time line of meal presentation and blood sampling. During meal-test sessions, test foods were consumed within 15 min by all rats. Blood samples were collected 10 min before food presentation and at multiple post-meal time points.

In the weeks prior to experimental testing, the rats were trained to consume 10 g of the test foods within 8–15 min after presentation to ensure prompt and complete consumption of test foods during subsequent test sessions. Ten to 15 days before the start of test sessions, each animal received a surgically implanted chronic jugular-vein catheter under deep anesthesia. These catheters enable repeated blood sampling without skin puncturing, at reduced stress levels and at volumes sufficient to determine several hormones in parallel (23). A 10-day post-surgical recovery period was scheduled according to guidelines approved by the Wageningen University animal care and use committee, after which all animals had regained their pre-operative body weight. Starting at 4 days post-surgery, the rats received daily liquid meal presentations to verify and reinstate their prompt and rapid consumption of food. The experimental phase of this study proceeded according to a within-subject, repeated-measurement study design. Rats received the individual test foods in a quantity of 10 g (11 kcal) during weekly sessions (Fig. 1). The order of presentation of the different foods was balanced across rats. Before each of the three test sessions, rats were food deprived for 18 h to establish stable baseline hormone levels. During the meal sessions the animals were presented with a bowl containing one of the test foods. Blood was collected 10 min before food presentation (baseline sample), and at 20, 40, 60, 120, and 180 min thereafter. Because of occluding catheters, no blood could be collected from two animals, thus reducing the number of subjects to 10. Blood parameters were selected for their relevance as physiological signals mediating post-meal satiety. Blood sampling times were selected to obtain an adequate view of the meal-induced response within a 3-h time window. Blood glucose levels were analyzed using a portable glucose meter (Accu-Check; Roche, Indianapolis, IN). The remaining blood was transferred to EDTA-containing tubes with protease inhibitors aprotinin (0.6 TIU/ml of blood; Phoenix Europe GmbH, Karlsruhe, Germany) and dipeptidyl-peptidase inhibitor (2.5 µl; Millipore, Darmstadt, Germany). Blood tubes were immediately put on ice and centrifuged at 1,600 g and 4°C within 1 h after blood collection, to isolate the plasma which was stored immediately at −80°C until assaying. Plasma levels were determined of the gastrointestinal hormones ghrelin, CCK, GLP-1, and PYY. Ghrelin is produced primarily in the stomach; its plasma levels correlate with hunger and are suppressed after meals (24). CCK, historically, the first-described gastrointestinal satiation peptide, is released from I-cells in the duodenum and proximal jejunum in response to all macronutrients, but most prominently by fats and proteins (25). To moderate the blood volume taken from rats, CCK was measured only at baseline and 60 and 120 min time points only. GLP-1 and PYY are produced in the small and large intestines; their blood levels correlate with satiation and rise in response to the intestinal-luminal presence of meal-related digesta (25, 26). Acute PYY release may underlie satiation and elevated GLP-1 levels have been found in humans after high protein diets (27). With the exception of CCK, commercially available ELISA kits were used to determine plasma levels of endogenous peptides according to the standard directions from the manufacturer). Insulin was analyzed by kit 80-INSRTU-E01 (Alpco Diagnostics; lower detection limit [LDL]: 0.1 ng/ml). Plasma ghrelin was measured by kit EK-031-31, which detects total ghrelin, that is, the combined octanoylated (bioactive) and des-octanoylated forms (28) in rats (LDL: 0.12 ng/ml). CCK was measured by a selective RIA method developed at the laboratory of Dr. J.F. Rehfeld, Department Clinical Biochemistry, University of Copenhagen, Denmark. GLP-1 plasma levels were determined with RIA FEK-028-11 (LDL 27.6 pg/ml), which detects the main gastrointestinally secreted, bioactive form of GLP-1 (GLP-1 (7–36)-amide) as well as its primary, inactive, metabolite (GLP-1-(9–36) amide, a commonly used marker of GLP-1 secretion by virtue of its slightly longer half-life in plasma (29, 30). PYY was measured by kit FEK-059-03 (LDL 16.2 pg/ml), which detects PYY(1–36) and PYY(3–36), the two main circulating, bioactive forms in rats and other mammals (31).

### Data analysis

For each blood parameter, in addition to analysis of the time course, two indices were calculated to capture the intensity of the test-meal-induced responses: DEV was the maximum post-meal deviation from baseline levels measured just before the meal, and area under the curve (AUC) was calculated according to the trapezoid method to reflect the integrated response all post-meal observations combined. Statistical analyses on these parameters were conducted using Excel (Microsoft, Redmond, USA) and SYSTAT 11.0 software (Systat, Chicago, IL, USA). Experimental effects were evaluated by parametric one-way, three-level repeated-measurement ANOVAs, with a threshold level for statistical significance set at *P*=0.05.

## Results

### *In vitro* simulation of gastric digestion of proteins

**Fig. 2:**
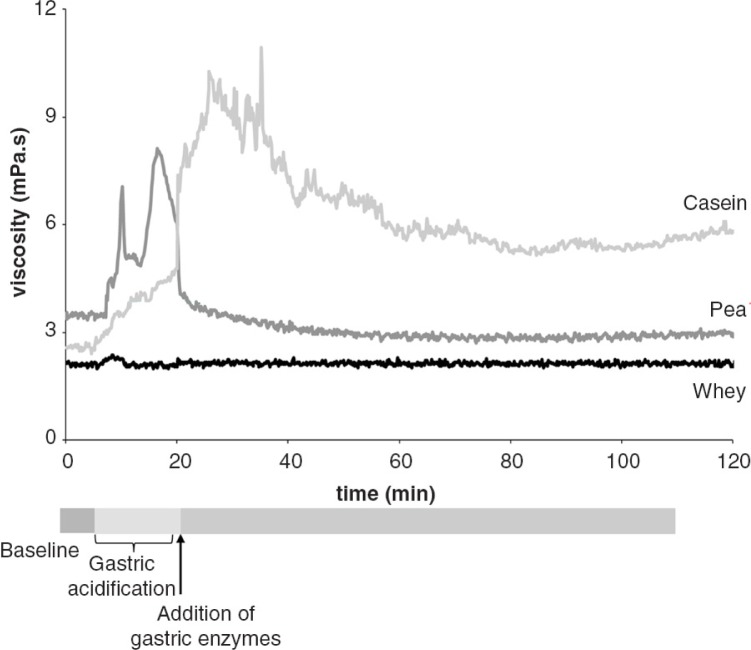
Two-hour viscosity profiles during simulated gastric digestion (SIMPHYD) of solutions of pea protein, bovine whey and casein. After a 5-min baseline measurement, acidification of the medium toward pH 1.5–2 was started and completed after circa 15 min. Then, gastric digestive enzymes were added to the medium.

The observed digestion kinetics were different for the three tested proteins (Fig. 2). For casein solutions, when the pH of the medium was lowered toward casein's isoelectric point, a rise in viscosity occurred that remained stable and elevated throughout the 2-h measurement period. Presumably, this behavior was related to the formation of a protein network by aggregation of casein molecules. *In vivo*, these fragments are broken down by the concerted action of gastrointestinal proteases and antral grinding. Upon addition of gastric digestive enzymes viscosity of the casein-containing medium declined, although it remained higher than that of whey and pea-protein solutions throughout the 2-h monitoring period. In contrast, and in line with expectations, whey protein displayed a relative stable low-viscosity profile, attributable to the absence of protein aggregation. Finally, digestion of pea protein was characterized by an increase of viscosity of the medium when the pH of the medium was brought down, approaching the isoelectric point of pea protein. This profile was probably related to solubility of pea protein, as confirmed by direct solubility measurements (data not shown) and particle-size measurements in pea-protein solutions at various pH levels. Observed pea-protein particle sizes ranged between 1.7 and 267 µm (median: 80 µm), which is considerably smaller than the 2-mm (i.e. 2,000 µm) threshold for prolonged gastric retention seen with slowly digestible proteins like casein.

### *In vivo* gastrointestinal hormone responses to protein meals

**Fig. 3:**
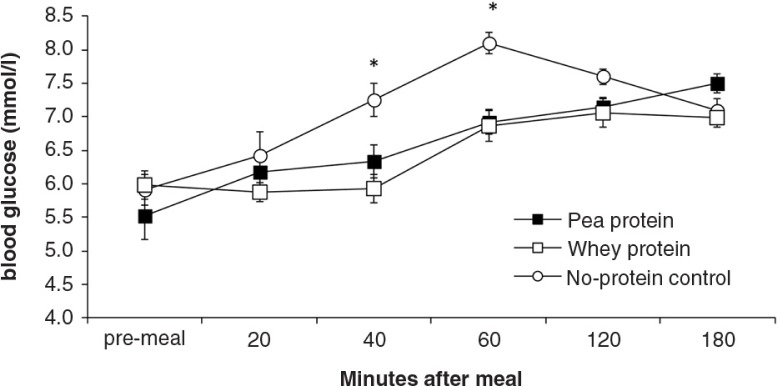
Blood glucose levels (mean±s.e.m.) in response to experimental meals. Between-meal differences occurred at 40 min (*F*(2,18)=7.8; *P*=0.004) and 60 min (*F*(2,18)=14.1; *P*<0.001). Rank order of blood glucose levels: WP, PP<NP (PP=pea protein; WP=whey protein; NP=non-protein control).

**Fig. 4:**
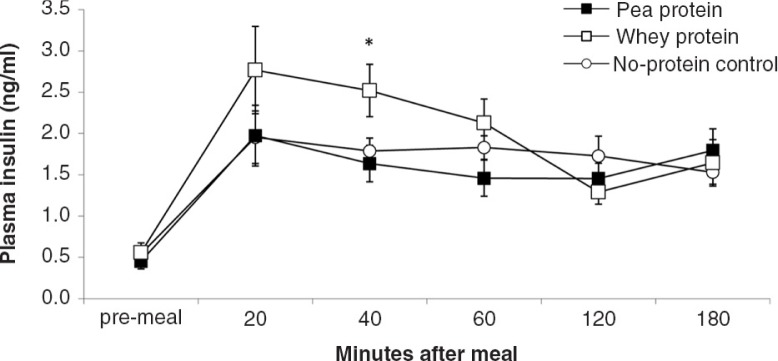
Plasma insulin levels (mean±s.e.m.) in response to experimental meals. Between-meal differences occurred at 40 min (*F*(2,18)=5.1; *P*=0.017). Rank order of plasma insulin levels at both time points: NP, PP<WP.

**Fig. 5:**
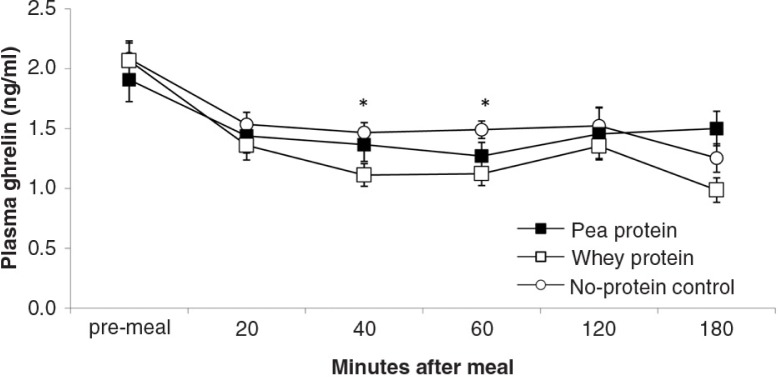
Plasma ghrelin levels (mean±s.e.m.) in response to experimental meals. Between-meal occurred at 40 min (*F*(2,18)=4.2; *P*=0.03) and 60 min (*F*(2,18)=4.2; *P*=0.03). Rank order of plasma ghrelin levels at both time points: WP<PP, NP.

**Fig. 6:**
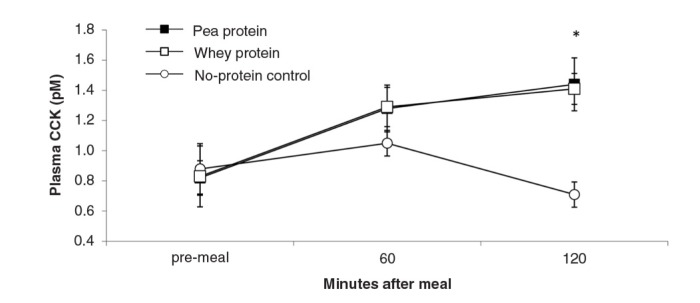
Plasma CCK levels (mean±s.e.m.) in response to experimental meals. Between-meal differences occurred at 120 min (*F*(2,18)=15.8; *P*<0.001). Rank order of plasma levels: NP<PP, WP.

**Fig. 7:**
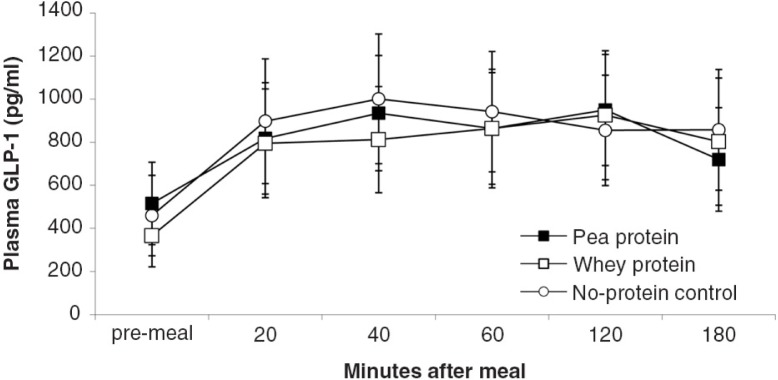
GLP-1 plasma levels (mean±s.e.m.) in response to experimental meals.

**Fig. 8:**
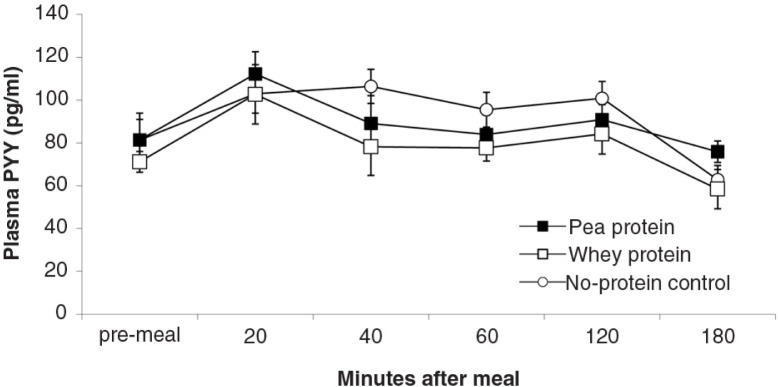
PYY plasma levels (mean±s.e.m.) in response to experimental meals.

During all sessions, all rats consumed the 10 g (11 kcal) test meals within 15 min after presentation. Figures 3–8 depict the time-dependent responses of blood parameters after the three test meals. Between-condition comparisons of maximum deviation from baseline (DEV) and integrated responses (AUC) are shown in Tables 2 and 3. Pea-protein and whey-protein meals similarly affected all blood parameters, except insulin and ghrelin, for which whey meals triggered a larger deviation from pre-meal baseline. Pea-protein and whey-protein meals stimulated CCK more strongly, indicated by larger DEV, AUC, and 2-h post-meal plasma CCK levels than did the non-protein (sucrose) meals. Ghrelin and insulin changed more strongly after whey- than by pea-protein or non-protein control, at 40 and 40/60 min, respectively; the integrated response size (AUC) for whey meals was larger only for ghrelin. The non-protein control meals, containing high concentrations of the glucose-containing disaccharide, sucrose, predictably increased the AUC of blood glucose response curve and the intensity at selected early time points (40 and 60 min post-meal).

**Table 2:** Maximum post-meal deviation of blood glucose, plasma insulin, and gastrointestinal hormone levels following the three test meals (*N*=10 rats)

	Pea protein	Whey protein	No protein	ANOVA
Blood glucose (mmol/l)	2.1±0.3	1.5±0.3	2.4±0.3	ns
Insulin (ng/ml)	1.9±0.3	2.7±0.4	2.0±0.2	ns
Ghrelin (ng/ml)	−0.8±0.2	−1.2±0.2	−0.9±0.2	*F*(2,18)=5.9; *P*=0.01^a^
CCK (pM)	0.7±0.2	0.8±0.2	0.2±0.2	*F*(2,18)=4.0; *P*=0.037^b^
PYY (pg/ml)	44±12	39±11	43±9	ns
GLP-1 (pg/ml)	577±111	619±136	582±131	ns

Significant differences are denoted by F statistics and *p*-values of one-way repeated-measurement ANOVAs. Rank order of intensity:

a WP<PP, NP

b NP<PP, WP by paired *t*-tests.

**Table 3:** Area under the curve of blood glucose, plasma insulin, and gastrointestinal hormones following the three test meals (*N*=10 rats)

	Pea protein	Whey protein	No-protein	ANOVA
Blood glucose (minute*mmol/l)	1,236±16	1,204±20	1,347±18	*F*(2,18)=20.8; *P*<0.001^a^
Insulin (minute*ng/ml)	276±31	323±37	303±179	ns
Ghrelin (minute*ng/ml)	258±24	226±15	270±16	ns
CCK (minute*pM)	148±15	142±12	111±5	*F*(2,18)=4.0; *P*=0.035^b^
PYY (minute*pg/ml)	15,700±1,142	14,239±1,438	16,756±896	ns
GLP-1 (minute*pg/ml)	151,188±41,340	149,956±48,440	157,280±48,118	ns

Significant differences are denoted by F statistics and *p*-value of one-way repeated-measurement ANOVAs. Rank order of intensity by paired *t*-test:

a PP, WP<NP

b NP<PP, WP.

## Discussion

The goal of the current studies was to more precisely characterize satiety-related properties of NUTRALYS^®^ pea protein during gastrointestinal processing in comparison with more extensively studied proteins of animal origin (whey and casein). Protein solubility and aggregation in an *in vitro* model of stomach digestion and 3-h post-ingestive hormonal responses in rats were examined. During stomach digestion, pea protein temporarily precipitated, in contrast to the fast-digestible protein, bovine whey, which remained in solution. The size of pea-protein precipitates was smaller (between 50 and 500 µm) than the quintessential slow-digestible protein, bovine casein. *In vivo* comparisons between isocaloric meals containing 35 energy% of pea-protein, whey-protein or isocaloric carbohydrate showed that 1) pea-protein and whey-protein meals induced similar plasma rises of the anorexigenic hormones CCK, GLP-1 and PYY; 2) at initial time points, whey-protein meals induced slightly more intense ghrelin and insulin plasma responses than pea-protein meals c) both pea-protein and whey-protein meals induced larger CCK responses than did the carbohydrate meal.

These results support the qualification of pea protein as an intermediate fast protein that moderately and transiently aggregates in the stomach and becomes intestinally available for satiety signaling slightly later than whey, the quintessential fast-digestible protein. Delayed intestinal bioavailability of pea protein compared to whey might partly explain the less intense early post-meal insulin and ghrelin responses. Prolonged gastric retention of aggregating pea-protein particles, such as described for casein (8) is unlikely to explain these findings, because the pea-protein aggregates had insufficient size (<<2 mm) to be blocked by the constricting pylorus. More likely, intestinal breakdown of the gastrically formed multi-molecular pea-protein particles may have required more prolonged action of intestinal proteases than that of single, dissolved whey molecules. Thus, while there may be significant breakdown of orally ingested pea protein within the stomach (18), it would be worthwhile to examine if systematically increasing the particle size of still intact pea protein emptied from the stomach could be used to evoke prolonged satiety signals from the small and large intestine.

The relevance of the *in vivo* data lies in the prominent role of gastrointestinal hormones as physiological mediators of appetite and satiety (24, 32) as also demonstrated in high-protein diets (27). CCK, GLP-1, and PYY are considered the primary anorexigenic gut peptides. We observed larger stimulation of CCK by pea- and whey-protein meals than by the carbohydrate (sucrose) control meals, confirming robust findings from human and animal studies (25, 33). Although CCK release has been linked to the speed by which proteins enter the small intestine (10), pea- and whey-protein meals had similar hormonal effects. Thus, bearing in mind the equal amounts of fat – the other CCK-releasing macronutrient – in the two test meals, pea protein and whey appear to be equally effective CCK releasers. Importantly, the current data do not allow conclusions about the role of CCK in acute protein-induced satiation. Rats consumed the presented 11-kcal test meals completely in all conditions, and CCK levels were not monitored during or immediately after meal presentation. In contrast, similar to findings in humans (33), the protein-specific elevation of CCK was observed 120-min post-meal, that is, when inter- rather than intra-meal mechanisms are activated.

Compared to CCK, the distribution of PYY and GLP-1 is spread out more widely along the small and large intestines. The observed rapid steady rise in post-prandial plasma levels of these gut peptides and the absence of a late rise suggest that the test meals acted via proximal-intestinal mechanisms, by direct contact of nutrients with peptide-hormone releasing cells and indirectly by duodenally activated release from distal areas, e.g. the ileum and colon (24, 34, 35). Assuming that protein breakdown into di- and tripeptides and amino acids is required to induce PYY and GLP-1 release, the equal hormonal effects of pea- and whey protein meals indicate similarly adequate small-intestinal bioavailability of pea- and whey-protein metabolites. The finding that the protein and non-protein sucrose meals triggered comparable PYY and GLP-1 responses is understandable, given the stimulatory effect of glucose (a metabolite of the used sucrose) on these two hormones (36, 37). Thus, in the current study, proteins did not show larger efficacy than isocaloric sucrose in triggering PYY and GLP-1, although the physiologic pathways involved may have differed.

Ghrelin was the only orexigenic hormone measured in this study. Its plasma levels correlate with meal-time hunger and are suppressed by nutrients through post-gastric mechanisms (26, 38). We observed an initial post-meal ghrelin suppression that was slightly stronger after whey protein than after pea-protein or sucrose control meals, but the integrated 3-h effect on ghrelin (AUC) was similar for all meals. While protein-induced ghrelin suppression has been described before (26, 28), the stronger acute responses to whey-protein compared to pea-protein test meals could be explained in two ways. First, as suggested by the results from our *in vitro* experiment, whey protein was more rapidly bioavailable in the gastrointestinal tract than was pea protein. Second, an additional insulin-dependent suppression of ghrelin is plausible (39), because the higher insulin levels after whey protein compared to pea-protein meal occurred in the same time window (40 and 60 min post-meal). The finding that the total integrated ghrelin response (AUC) over 3 h was similar for two proteins indicates that such a mechanism – if it contributed – was limited to initial stages after the meal.

Post-meal dynamics of blood glucose and plasma insulin may support metabolic health, but their role in satiety or body weight is still under debate (40–42) . We observed a predicted rise in blood glucose after all three (sucrose-containing) test meals, with the largest AUC for the non-protein control meal, which had the highest sucrose content. Pea- and whey-protein likely increased blood glucose and insulin levels by additional post-absorptive mechanisms, that is, via hepatic and/or intestinal gluconeogenesis, which is typically pronounced after fast-breaking meals and insulinotropic action of absorbed amino acids (26). The stronger rise in insulin after whey-protein than after pea-protein meals at 40 min post-meal may be attributed to the higher level of branched-chain amino acid in whey (42). This stronger effect of whey on insulin was transient and 3-h integrated AUC for both insulin and blood glucose was similar for the two proteins. Clearly, the different insulin responses to the two protein meals cannot be attributed to an incretin effect by GLP-1 (43), because levels of the latter hormone were affected similarly by all test meals. Because the current data were derived from single-meal trials, long-term predictions of comparative efficacy of pea- vs. whey-protein related to glucose regulation (e.g. insulin resistance) would require longer dietary interventions and more tightly controlled procedures (i.e. glucose-clamp protocols). Although the similar hormonal responses to protein- and sucrose-rich meals we observed (except for CCK) are at odds with previous studies (e.g. (10, 28)), they are not unique. Smeets et al. (44), who have tested dairy protein in humans, found no differences in ghrelin, GLP-1, and PYY responses after high- and low-protein meals.

The direct application of the current results are constrained by the fact that dietary proteins are usually embedded in mixed-nutrient meals and post-meal metabolic effects are triggered by a joint action of different macronutrients via multiple physiological mechanisms (8, 10). Our data do suggest that whole or partial dietary replacement of animal by pea protein does not weaken gastrointestinal satiety signaling, despite pea protein's different amino-acid composition and altered gastrointestinal processing.
